# A New Hybrid BFOA-PSO Optimization Technique for Decoupling and Robust Control of Two-Coupled Distillation Column Process

**DOI:** 10.1155/2016/8985425

**Published:** 2016-10-11

**Authors:** Noha Abdelkarim, Amr E. Mohamed, Ahmed M. El-Garhy, Hassen T. Dorrah

**Affiliations:** ^1^Department of Electronics, Communications and Computers, Faculty of Engineering, Helwan University, 1 Sherif Street, Helwan, Cairo 11792, Egypt; ^2^Department of Electrical Engineering, Faculty of Engineering, Cairo University, University Street, Giza 12316, Egypt

## Abstract

The two-coupled distillation column process is a physically complicated system in many aspects. Specifically, the nested interrelationship between system inputs and outputs constitutes one of the significant challenges in system control design. Mostly, such a process is to be decoupled into several input/output pairings (loops), so that a single controller can be assigned for each loop. In the frame of this research, the Brain Emotional Learning Based Intelligent Controller (BELBIC) forms the control structure for each decoupled loop. The paper's main objective is to develop a parameterization technique for decoupling and control schemes, which ensures robust control behavior. In this regard, the novel optimization technique Bacterial Swarm Optimization (BSO) is utilized for the minimization of summation of the integral time-weighted squared errors (ITSEs) for all control loops. This optimization technique constitutes a hybrid between two techniques, which are the Particle Swarm and Bacterial Foraging algorithms. According to the simulation results, this hybridized technique ensures low mathematical burdens and high decoupling and control accuracy. Moreover, the behavior analysis of the proposed BELBIC shows a remarkable improvement in the time domain behavior and robustness over the conventional PID controller.

## 1. Introduction

Controller design for multi-input multiple-output (MIMO) systems is of significant importance, as they constitute the majority of physical systems. However, the design of such controllers is confronted with the challenge to overcome the influence of the nested interrelationship between system inputs and outputs. Based on the level of interrelationship between system inputs and outputs, the control structure is to be selected. The systems, which are characterized with moderate interaction between control loops, can be controlled with the decentralized control structure so that a single controller is assigned for each control loop [1–5]. On the other hand, a centralized controller has to be designed for systems with higher level of interrelationship between control loops [6–9], so that a central matrix of controllers is allocated for the whole MIMO system. In spite of this, the centralized controllers are not widely utilized due to their high complexity. Alternatively, the issue of high interrelationship can be alleviated by decoupling the MIMO system into several relatively independent single-input single-output (SISO) control loops [10–15]. Accordingly, the multivariable process can be controlled based on independent loop structure. In this paper, the decoupled control structure is utilized for controlling the presented MIMO system. For the decoupler to be designed, the adequate input-output pairings are primarily determined through the evaluation of the system relative gains [16–19]. Accordingly, the decoupling network has to be designed, so that the interaction between control loops is minimized [20, 21]. For control purposes, conventional control schemes as PID can be utilized [22–24]. It is to mention that the nonlinearity and the model imprecision that characterize the majority of physical systems reduce the robustness and accuracy of such controllers.

Control design approaches based on intelligent algorithms as fuzzy logic, neural network, and genetic algorithms are of increasing spread due to their proven ability to overcome system model uncertainty [25–27]. The computational model of emotional learning in mammalian brain, which is introduced in [28, 29], inspired a new learning algorithm. Afterwards, this learning technique is deployed in the system control design presenting the novel Brain Emotional Learning Based Intelligent Controller (BELBIC) [30]. Thus, the Brain Emotional Learning Based Intelligent Controller (BELBIC) has shown robustness in the control of the nonlinear and uncertain systems such as Van Der Pol oscillator, Duffing forced oscillator and automatic self-balancing scale [31], washing machine [32], microheat exchanger [33], switched reluctance motor [34], unmanned aerial vehicle [35], path tracking of a vehicle [36], two-coupled distillation column process [37], multiple-area power systems [38], and continuous stirred tank reactor [39, 40].

The parameterization of the decoupling compensation network and the BELBIC are still open research topics. Currently, the detailed analytical methods used in purpose of the parameterization of the decoupling compensation network cost excessive mathematical burdens. Regarding the parameterization of BELBIC, the trial and error is widely used for controller parameters estimation. Thus, the utilization of various optimization techniques based on artificial intelligence introduces an efficient alternative in both cases [11, 37].

The utilization of the biologically inspired algorithms as Ant Colony Optimization (ACO) [41], Genetic Algorithm (GA) [42], and Particle Swarm Optimization (PSO) [43, 44] in optimization problems constitutes currently a promising solution. Recently, the new evolutionary computation technique depending on the behavior of foraging of* E. coli* bacteria, which is named as Bacterial Foraging Optimization Algorithm (BFOA), is proposed by [45]. This technique has been successfully deployed in many applications as power systems [46–49], stock market prediction [50, 51], and design of PI/PID controllers [52, 53]. The key drawback of the BFOA is the delay in reaching the global solution because it is based on random searching directions. For this delay to be overcome, the BFOA is integrated with the Particle Swarm Optimization (PSO) technique, introducing the Bacterial Swarm Optimization (BSO) algorithm [54]. One of the major characteristics of PSO technique, which is inherited to the BSO algorithm, is the idea of velocity updating. Accordingly, the process of searching for the global solution in the BSO technique depends on the individual and global best positions concurrently. As discussed in [55–58], The Bacterial Swarm Optimization (BSO) algorithm grants better performance in determining the optimum solution compared with PSO and BFOA algorithms.

This research paper aims to investigate the feasibility of applying the Bacterial Swarm Optimization (BSO) algorithm in the field of MIMO control system. As an example for MIMO system the two-coupled distillation column is to be studied. Primarily, the mathematical model of the system is decoupled into several independent loops. The parameters of the decoupling compensation network are determined based on the summation minimization of the integral time-weighted squared outputs (ITSOs) of unpaired outputs regarding a particular input. In this regard, the BSO technique is utilized. On the other hand, an optimal BELBIC is designed for each decoupled control loop through the summation minimization of the integral time-weighted squared errors (ITSEs) of all loops using BSO as well. For the comparison purpose the PID controller is also implemented for the same application, so that the strength of each of considered control structures can be studied.

## 2. Two-Coupled Distillation Column Process

As formerly stated, the two-coupled distillation column is handled in this paper as an example of MIMO system. In this section, the physical system is discussed. Primarily, the function of the system and its components are presented. Afterwards, the system mathematical modeling and decoupling are handled.

### 2.1. System Description

Distillation units are mainly utilized for the separation of fluid mixture components. The distillation column major components can be listed as follows:

- A vertical shell in which the separation of fluid substances is accomplished
- A cascade of trays for improving component separation
- A reboiler for maintaining the required heat energy for the distillation process
- A condenser for liquefying the vapor leaving the column
- A reflux drum in which portion of the condensed liquid is recycled back to the vertical shell.

**Figure 1:**
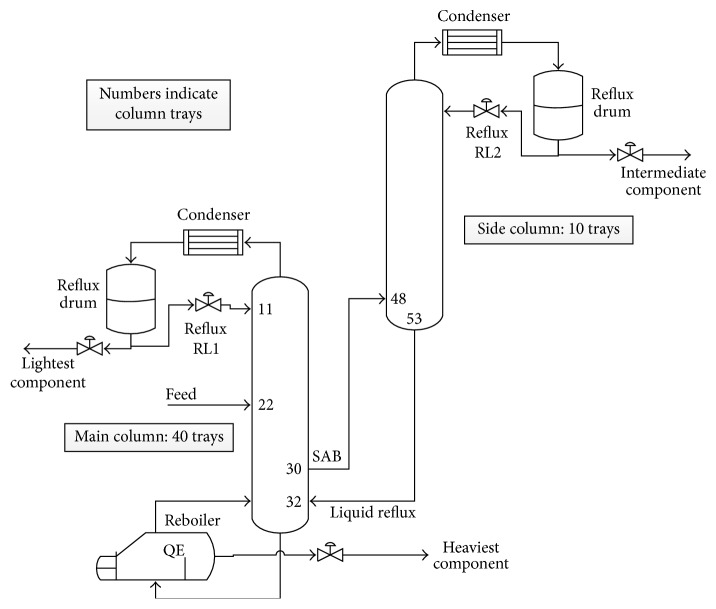
The two-coupled distillation columns process.

The physical process considered in this research comprises two-coupled distillation columns as shown in Figure 1. Thereby, ternary petrochemical mixtures can be separated. While the main glass column is composed of 40 bubble cap trays excluding those of the boiler and condenser, the side glass column contains 10 bubble cap trays. At the 22nd stage, the system intake port is located, at which the fluid mixture is to be fed. The three separated fluid components are to be extracted from the process, so that the heaviest and the lightest components can be provided from the bottom and the top of the main column, respectively, and the intermediate component is derived from the top of the side column.

### 2.2. Mathematical Modeling of the Two-Coupled Distillation Column

Primarily, the manipulated variables are to be determined. Thus, the selected manipulated variables are the heat input to the reboiler (QE), the vapor flow rate in the vapor transfer line (SAB), the reflux ratio in the main column (RL1), and the reflux ratio in the second column (RL2) [59]. Regarding the system technical constraints, the input heat energy rate QE and the vapor flow rate SAB have to not exceed the values 8.2 KW and 3.95 m^3^/h, respectively, in order to avoid flooding of the smaller side column [59]. Secondly, the controlled values are selected to be the fluid temperature at four different stages. The selection of these stages is based on the following requirements:

- The temperatures should be sensitive to the major disturbances as changes in feed rate and feed concentrations.
- The temperatures should exhibit a fairly linear behavior.
- They should be a good indication of product quality.

Accordingly, the temperature at the 11th, 30th, 34th, and 48th trays are chosen to form the controlled variables [59]. The mathematical model of realistic two-coupled distillation column, mentioned in [59], is to be used in this research. The transfer function matrix of the considered MIMO system is stated in (1).
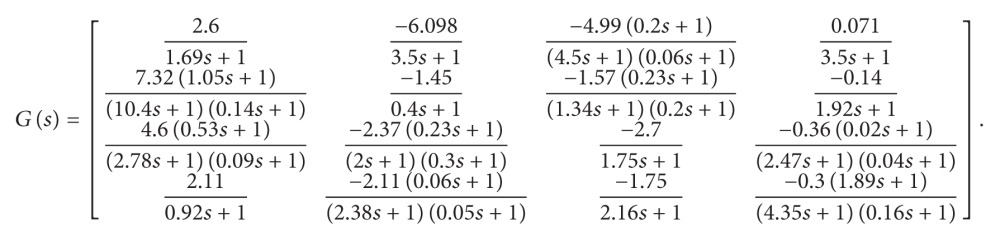
(1)


As shown in the transfer function matrix, the physical system is characterized by the high interrelationship between all system inputs and outputs. As discussed in the introduction, the control structure adopted in this paper is based on the mathematical model decoupling, so that each output is independently controlled by a single input, forming four independent SISO control loops.

Primarily, the noninteracting design is preceded by a relative gain analysis to determine the most suitable input-output pairings. In this regard, the relative gain array (RGA) method is used. Afterwards, a decoupling compensation network is designed for the reduction of residual interactions [11].

#### 2.2.1. The Relative Gain Array

The RGA matrix for *N* × *N* system represented in (2) is used for the valuation of the input influences on each system output [16–19], so that output “*i*” is to be paired with input “*j*” for which *γ*_*ij*_ is a positive value and as close to unity as possible.

(2) RGAN×NGs=0·∗Gs=0−T=γ11γ12⋯γ1Nγ21γ22⋯γ2N⋮⋮⋮γN1γN2⋯γNN,

where *G*(*s*) is the transfer function matrix of the MIMO process and “·*∗*” operator implies element by element multiplication.

#### 2.2.2. Decoupling Compensation Network Design Procedure

**Figure 2:**
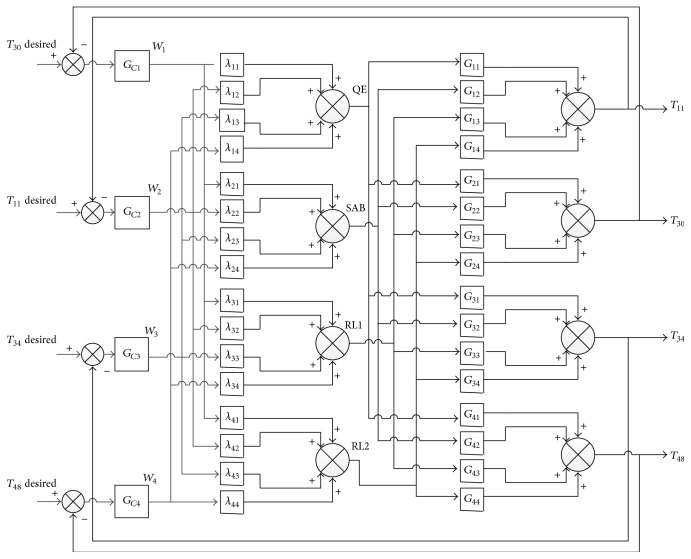
The decoupled MIMO system including controllers.

The steady state decoupling compensation matrix illustrated in (3) is to be integrated into the given MIMO system in the way demonstrated in (4), so that interrelationships between each input and unpaired outputs is minimized [20, 21]. In this paper, the simplest form of the decoupling compensation matrix introduced in the previous study [11], which has unity diagonal elements, is replaced with the general form presented in (3). This modification introduces a remarkable improvement in the system response as discussed in Section 5.  Figure 2 illustrates the decoupling compensation network scheme integrated with the required controllers.

(3) Λss=λ11λ12λ13⋯λ1N−1λ1Nλ21λ22λ23⋯λ2N−1λ2N⋮⋮⋮⋮⋮λN1λN2λN3⋯λNN−1λNN,

(4) Ys=GsΛssWs,

where [*Y*(*s*)] is the output vector, [*W*(*s*)] is the input vector, [*G*(*s*)] is the transfer function matrix of the MIMO process, and [Λ_ss_] is the steady state decoupling compensation matrix of MIMO process.

#### 2.2.3. Fitness Function Design

As discussed in the former two sections, the determination of the adequate system input/output pairing as well as the elements' values of the decoupling compensation matrix define the effectiveness of interrelationship minimization between system inputs and outputs. The selection of the proper input/output pairing is already tackled by the relative gain array concept. For the elements' values of the decoupling compensation matrix to be estimated using optimization techniques, the fitness functions have to be designed that minimize the interrelationship between system inputs and their unpaired outputs.

The four commonly used performance criteria for fitness function design are the integral absolute error (IAE), integral squared error (ISE), integral time-weighted squared error (ITSE), and Integral time-weighted absolute error (ITAE). It's to mention that in the context of decoupling system design, the performance criteria are concerned with the minimization of the system outputs towards the unpaired inputs rather than control system error. Accordingly, the corresponding criteria are integral absolute output (IAO), integral squared output (ISO), integral time-weighted squared output (ITSO), and Integral time-weighted absolute output (ITAO). Although the criteria ISO and IAO grant less overshoot in the system dynamics, none of these criteria is adopted in this research due to the long settling time [56]. This drawback could be overcome by utilizing the ITAO. However, this criterion is not used in this research for the difficulty of its analytical tracking [60]. Thus, the ITSO performance criterion is employed in this work for ensuring the minimization of settling time without being confronted with unnecessary analytical complications.

In the former research [11], the decoupling compensation matrix elements are estimated by applying the PSO technique, so that the integral squared outputs (ISOs) of unpaired outputs with respect to a specific input are minimized. On the other hand, the proposed technique in this paper is based on the minimization of the integral time-weighted squared outputs (ITSOs) by utilizing the BSO technique.

The integral time-weighted squared outputs (ITSOs) for each input are calculated such that

 if *W*_1_(*s*) = 1/*s* and *W*_2_, *W*_3_,…, *W*_*N*_ are zeroes, then
  (5) $$\begin{array}{c} ITSO_{11}=\overset{\infty }{\underset{0}{\int }}t{\left\{\;Y_{1}\;\right\}}^{2}\cdot dt,\\ ITSO_{21}=\overset{\infty }{\underset{0}{\int }}t{\left\{\;Y_{2}\;\right\}}^{2}\cdot dt,\\ \vdots \\ ITSO_{N1}=\overset{\infty }{\underset{0}{\int }}t{\left\{\;Y_{N}\;\right\}}^{2}\cdot dt \end{array}$$
 and if *W*_2_(*s*) = 1/*s* and *W*_1_, *W*_3_,…, *W*_*N*_ are zeroes, then
  (6) $$\begin{array}{c} ITSO_{12}=\overset{\infty }{\underset{0}{\int }}t{\left\{\;Y_{1}\;\right\}}^{2}\cdot dt,\\ ITSO_{22}=\overset{\infty }{\underset{0}{\int }}t{\left\{\;Y_{2}\;\right\}}^{2}\cdot dt,\\ \vdots \\ ITSO_{N2}=\overset{\infty }{\underset{0}{\int }}t{\left\{\;Y_{N}\;\right\}}^{2}\cdot dt. \end{array}$$
 Consecutively, if *W*_*N*_(*s*) = 1/*s* and *W*_1_, *W*_2_,…, *W*_*N*−1_ are zeroes, then
  (7) $$\begin{array}{c} ITSO_{1N}=\overset{\infty }{\underset{0}{\int }}t{\left\{\;Y_{1}\;\right\}}^{2}\cdot dt,\\ ITSO_{2N}=\overset{\infty }{\underset{0}{\int }}t{\left\{\;Y_{2}\;\right\}}^{2}\cdot dt,\\ \vdots \\ ITSO_{NN}=\overset{\infty }{\underset{0}{\int }}t{\left\{\;Y_{N}\;\right\}}^{2}\cdot dt. \end{array}$$

Thus the fitness function for specific input *W*_*j*_ can be described as follows:

(8) $$\begin{array}{c} Fitness_{j}=\overset{N}{\underset{i=1}{\sum }}ITSO_{ij},\;j=1,2,3,\ldots ,N,\;\;i\neq q, \end{array}$$

where *i* is the specific output subscript, *j* is the specific input subscript, and *q* is the subscript of the output that has been paired with input *j*.

## 3. Controlling of Two-Coupled Distillation Column Process

In this section, the proposed BELBIC and the conventional PID controller are handled regarding the control structure.

### 3.1. Brain Emotional Learning Based Intelligent Controller (BELBIC) Model

As formerly stated, the considered MIMO system is to be split into several decoupled SISO systems with the minimum possible interrelationship between them. Thus, each of these systems can be independently controlled. In this research, the BELBIC adaptive control structure is utilized for each single control loop. This control structure is based on the functional model of brain emotional learning introduced by [28, 29]. Over the past decade, this control scheme has proven its robustness in many complex control applications [31–40]. Apart from the application in control systems, the computational model of brain emotional learning in its discrete and continuous form is discussed here, respectively. Afterwards, the methodology of the proposed BSO-BELBIC scheme, which assigns one BELBIC for each decoupled loop, is presented.

**Figure 3:**
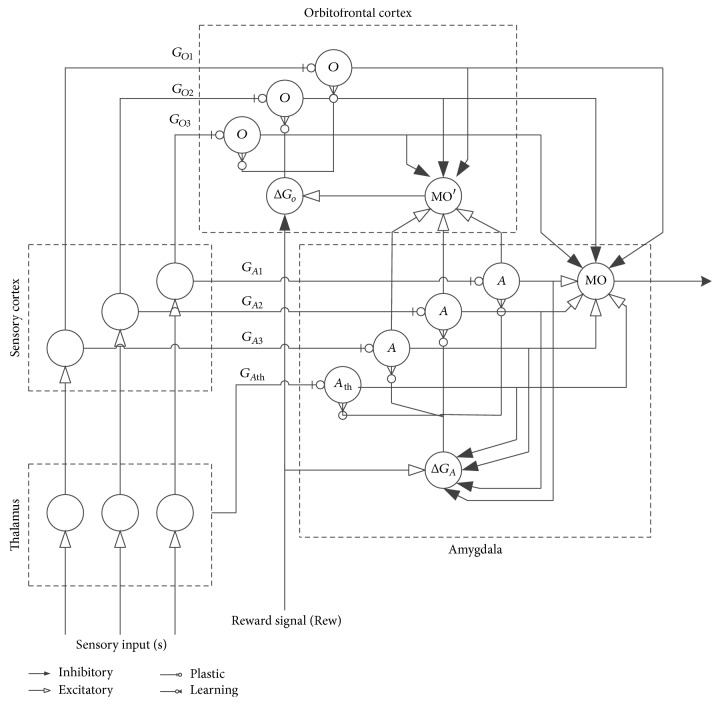
Graphical depiction of the brain emotional learning (BEL) process [28, 29].

The emotional learning computational model designed by [28, 29] is graphically illustrated in Figure 3. In mammalian brains, the emotional learning process occurs in a part of the brain called the limbic system, which consists of four main components corresponding to the amygdala, orbitofrontal cortex, thalamus, and the sensory cortex. As shown in the figure, sensory input signals are primarily passed to the thalamus model, which relay the sensory information from the peripheral sensory systems to the sensory cortices. Moreover, the sensory input maximum value is passed directly to the amygdala for the fast response to be insured. Then, the available sensory data are to be processed by sensory cortex model. Hence, highly analyzed data are to be sent to the amygdala and orbitofrontal cortex models. The emotional evaluation of stimuli and the formulation of long-term memories are carried out by the amygdala. Finally, the orbitofrontal cortex is supposed to inhibit inappropriate responses from the amygdala.

The outputs of the model two major components amygdala and orbitofrontal cortex are described in (9) and (10), respectively. The feedback element MO′ is defined in (11) as the subtraction of the orbitofrontal cortex inhibitory outputs (*O*_*i*_) from the summation of amygdala nodes (*A*_*i*_) excluding *A*_th_ node. As illustrated in relation (12), the output (MO) of the brain emotional learning model (BEL) constitutes the subtraction of the orbitofrontal cortex inhibitory outputs (*O*_*i*_) from the summation of amygdala nodes (*A*_*i*_) including the *A*_th_ node.

(9) Ai=SiGAi,

(10) Oi=SiGOi,

(11) MO′=∑iAi−∑iOiexcluding  Ath  node.,

(12) MO=∑iAi−∑iOiincluding  Ath  node.,

where *S*_*i*_ forms the *i*th sensory input, and *G*_*A*_ and *G*_*O*_ are the plastic connection weights of the amygdala and orbitofrontal cortex, respectively. These plastic connection weights are responsible for the emotional change towards specific object characteristics. Thus, they constitute the adaptive component in the model structure, as their values are to be updated continuously. While the formulas (13) and (14) represent the discrete form for the change in plastic connection weights, (15) and (16) constitute the continuous one. It is to mention that the continuous form is utilized in this research.

(13) ΔGAi=αSimax⁡0,Rew−∑jAj,

(14) ΔGOi=βSiMO′−Rew,

(15) dGAidt=α·Si·Rew−Ai,

(16) dGOidt=β·Si·Ai−Oi−Rew,

where *α* and *β* are learning rate constants, and the symbol Rew forms the reward signal. The operator “max” in the formula (13) is the responsible for maintaining the monotonic learning change of amygdala. This characteristic models the incapability of the amygdala to unlearn the formerly learned emotions [28, 29]. In the continuous form, the operator “max” is eliminated for analytical simplicity [61].

**Figure 4:**
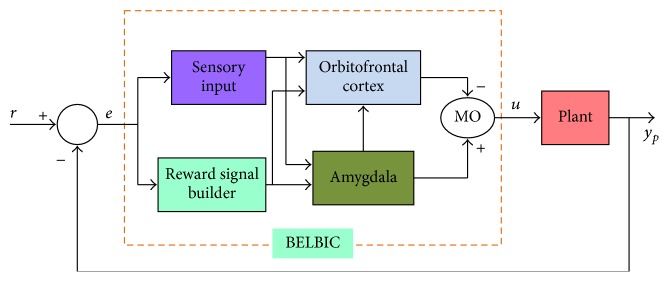
Control system configuration using BELBIC.

The BELBIC internal structure as well as its interface with the controlled physical plant is illustrated in Figure 4. As shown, sensory input block as well as the reward signal builder manipulates orbitofrontal cortex and amygdala based on the control error signal according to (17) and (18), respectively.

(17) S=K·e,

(18) Rew=Kp·e+Ki·∫e·dt+Kd·dedt,

where *K*, *K*_*p*_, *K*_*i*_, and *K*_*d*_ besides the learning rate constants (*α* and *β*) constitute the controller parameters, which characterize the controlled dynamic system behavior.

The BELBIC parameterization is one of the current challenges confronting such a novel control structure. In this regard, the utilization of optimization techniques like Particle Swarm Optimization (PSO) has shown a robust behavior [37]. In the frame of this research, the feasibility of applying the Bacterial Swarm Optimization (BSO) algorithm regarding the tuning process of BELBIC parameters is to be investigated. As mentioned in the previous section, the integral time-weighted squared errors (ITSEs) of all control loops are selected to be minimized rather than the integral-squared-errors (ISEs) for its faster settling response. Accordingly, the fitness function is represented in (19).

(19) Fitness  function=∫0∞tT30  desired−T302·dt+∫0∞tT11  desired−T112·dt+∫0∞tT34  desired−T342·dt+∫0∞tT48  desired−T482·dt.

Twenty-four parameters (six for each loop) should be tuned simultaneously with the aim of minimizing the fitness function.

### 3.2. PID Control

**Figure 5:**
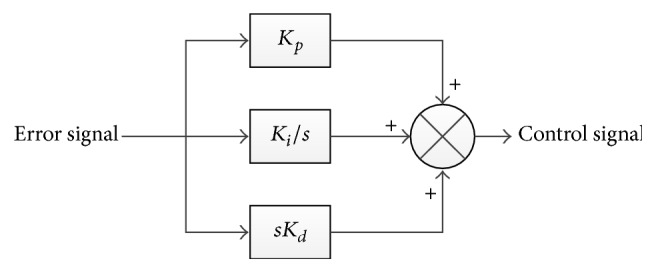
Block diagram of the conventional PID controller.

The main terms that constitute the conventional PID controller are the proportional, the integral, and the derivative terms. The three terms are added to each other as shown in Figure 5. The transfer function of the conventional PID controller is stated in (20).

(20) $$\begin{array}{c} G_{c}\left(\;s\;\right)=K_{p}+\frac{K_{i}}{s}+sK_{d}, \end{array}$$

where *K*_*p*_, *K*_*i*_, and *K*_*d*_ are proportional gain, integral gain, and a derivative gain, respectively.

The Bacterial Swarm Optimization (BSO) algorithm is utilized regarding the parameterization of PID controllers with the same policies as BELBICs.

## 4. Bacterial Swarm Optimization Algorithm

Bacterial Swarm Optimization Algorithm (BSO) forms a hybrid between two efficient optimization techniques. These algorithms are the Particle Swarm Optimization (PSO) and the Bacterial Foraging Optimization Algorithm (BFOA).

PSO is a stochastic optimization approach inspired from the behavior of the flock of birds, insects, and fish. Every single particle in the search space adjusts its own direction based on its own experience as well as the experience of the most successful particle in the swarm [43, 44]. Nevertheless, the optimization technique based on the PSO algorithm may lead to entrapment in local optimum solution rather than catch the global one due to the rapidity and simplicity of the algorithm [62].

On the other hand, BFOA is a new bio-inspired algorithm depending on foraging behavior of* Escherichia coli* (*E. coli*) bacteria [45]. Bacteria have the tendency to group around the nutrient-rich regions by the activity named chemotaxis. The bacteria which fail to reach nutrient-rich areas may die due to the nutrient lack. However, the ones that survived reproduce the next generation in nutrient-rich areas. Once the current living environment becomes inconvenient to the bacteria, it tends to disperse randomly to search for an alternative environment. Consequently, the optimization technique that simulates the foraging behavior of these bacteria requires a long time for achieving the global optimum solution due to the dependence on random search directions [57].

The time consumed by BFOA finding the global optimum solution can be reduced by granting the* E. coli* bacteria the ability of exchanging social information. This ability is inherited from the PSO technique forming the BSO algorithm. Therefore, BSO algorithm requires less time for the optimum solution determination, while maintaining the BFOA ability in finding a new solution with elimination and dispersal. Thus, the BSO algorithm solves the insufficient scattering problem that confronted the PSO algorithm. Furthermore, the chemotaxis step of BSO technique safeguards against the PSO shortcoming regarding the weak search ability.

**Figure 6:**
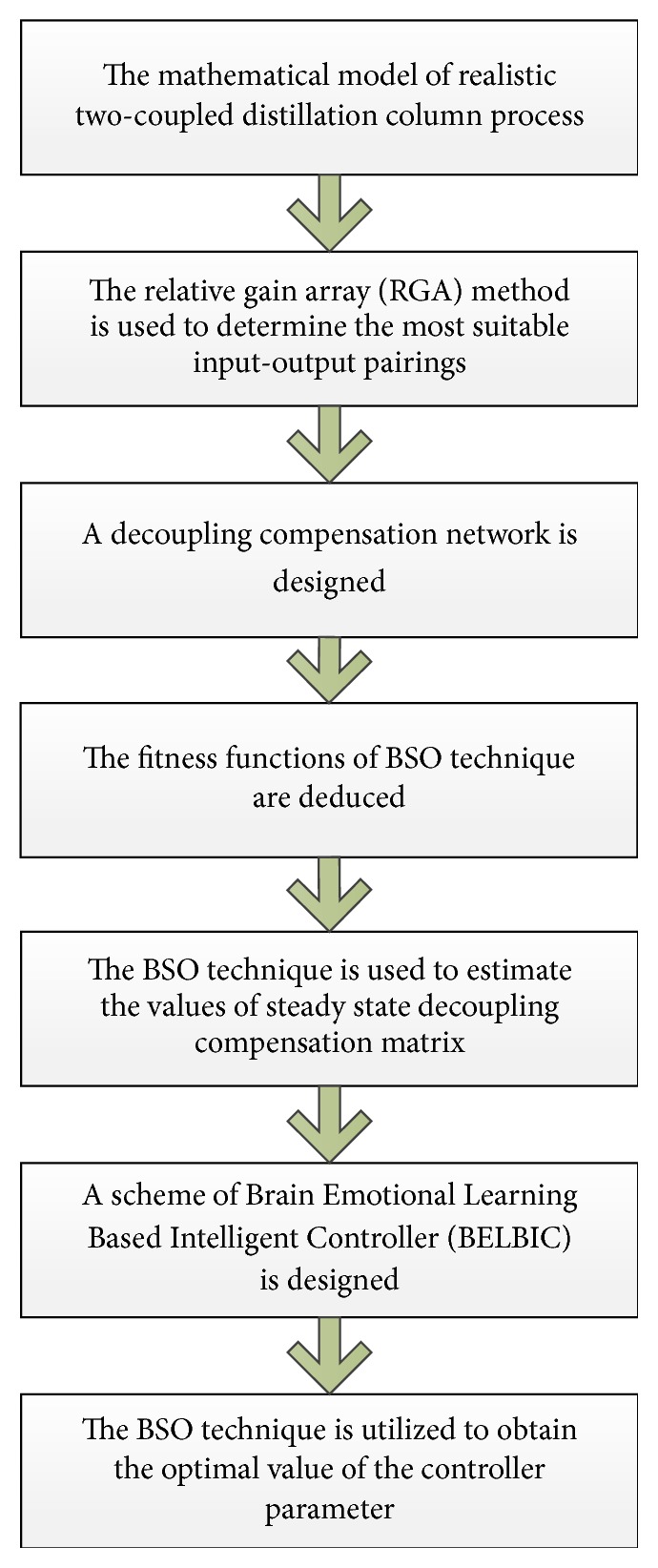
Process chart for the main steps of applying the proposed technique on the case study.

**Figure 7:**
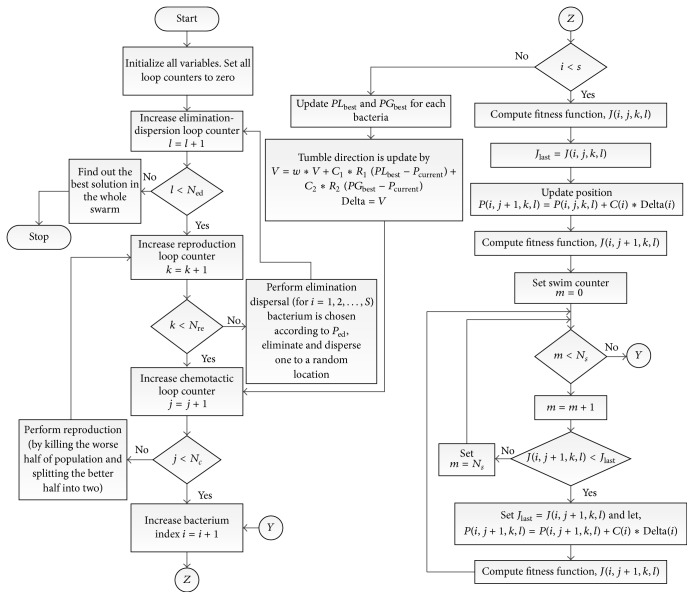
Flowchart of BSO algorithm.

As mentioned, the BSO technique is utilized for the parameterization of the decoupling compensation network as well as the BELBIC. As an overview, the procedure of applying the proposed technique on the case study is demonstrated in the process chart in Figure 6. Afterwards, the basic flowchart of the BSO algorithm is presented in Figure 7. Moreover, the pseudocode of the BSO algorithm is stated delivering more details.

The pseudocode of the BSO algorithm is as follows.

**Step 1 .**

The initialization of all stated parameters: *p*, *S*, *S*_*r*_, *N*_*c*_, *N*_*s*_, *N*_re_, *N*_ed_, *P*_ed_, *C*(*i*)  (*i* = 1,2,…, *S*), Delta, *w*, *C*_1_, *C*_2_, *R*_1_, and *R*_2_, where

(i) *p* is dimension of search space,
(ii) *S* is the number of bacteria,
(iii) *S*_*r*_ is the number of bacteria splits per generation,
(iv) *N*_*c*_ is the number of chemotactic steps,
(v) *N*_*s*_ is the limits of the length of a swim,
(vi) *N*_re_ is the reproduction steps number,
(vii) *N*_ed_ is the amount of elimination-dispersal events,
(viii) *P*_ed_ is the elimination-dispersal probability,
(ix) *C*(*i*) is the bacteria step size length,
(x) Delta is the direction of each bacteria,
(xi) *w* is the weight of inertia,
(xii) *C*_1_ is the weight of local information,
(xiii) *C*_2_ is the weight of global information,
(xiv) *R*_1_, *R*_2_ are two Random numbers.

**Step 2 .**

Loop of elimination and dispersal: *l* = *l* + 1.

**Step 3 .**

Loop of reproduction: *k* = *k* + 1.

**Step 4 .**

Loop of chemotaxis: *j* = *j* + 1.

*Substep  4.1*. For *i* = 1,2,…, *S* each bacterium *i* moves a chemotactic step as follows.

(a) Calculate cost function, *J*(*i*, *j*, *k*, *l*).
(b) Let *J*_last_ = *J*(*i*, *j*, *k*, *l*) to save the current value of the cost function in order to be able to compare it with the one determined in the next swim.
(c) Let *J*_local_(*i*, *j*) = *J*_last_; the better cost per each bacteria is going to be chosen to be the local best *J*_local_.
(d) Update position *P*(*i*, *j* + 1, *k*, *l*) = *P*(*i*, *j*, *k*, *l*) + *C*(*i*)*∗*Delta(*i*).
(e) Calculate cost function, *J*(*i*, *j* + 1, *k*, *l*).
(f) Swim
  (i) Let *m* = 0 (counter for swim length).
  (ii) While *m* < *N*_*s*_ (if have not climbed down too long).
    (1) Let *m* = *m* + 1.
    (2) If *J*(*i*, *j* + 1, *k*, *l*) < *J*_last_ (if doing better), let *J*_last_ = *J*(*i*, *j* + 1, *k*, *l*) and let
      (21) $$\begin{array}{c} P\left(\;i,j+1,k,l\;\right)=P\left(\;i,j+1,k,l\;\right)+C\left(\;i\;\right)*Delta\left(\;i\;\right) \end{array}$$
     and use this *P*(*i*, *j* + 1, *k*, *l*) to calculate the new *J*(*i*, *j* + 1, *k*, *l*).
    (3) For each bacteria estimate the current position and local cost
      (22) Pcurrenti,j+1=Pi,j+1,k,l,Jlocali,j+1=Ji,j+1,k,l.
    (4) Else, let
      (23) Pcurrenti,j+1=Pi,j+1,k,l,Jlocali,j+1=Ji,j+1,k,l,m=Ns.
    (5) While statement end.
(g) Go to next bacterium (*i* + 1) if *i* ≠ *S* (i.e., go to (b) to execute the next bacterium).

*Substep  4.2*. For each bacteria estimate the local best position (*PL*_best_) and global best position (*PG*_best_).

*Substep  4.3*. For each bacteria estimate the new direction as

(24) V=w∗V+C1∗R1PLbest−Pcurrent+C2∗R2PGbest−Pcurrent,Delta=V.

**Step 5 (if *j* < *N*_*c*_, go to Step 4).**

In this situation keep on chemotaxis since the life of the bacteria is not terminated.

**Step 6 (reproduction).**

*Substep  6.1*. For *k* and *l* and for each *i* = 1,2,…, *S*, the health of the bacterium *i* is given by

(25) $$\begin{array}{c} J_{health}^{i}=\overset{N_{c}+1}{\underset{j=1}{\sum }}J\left(\;i,j,k,l\;\right). \end{array}$$

Sort bacteria cost *J*_health_ in ascending order (greater cost indicates lower health).

*Substep  6.2*. The *S*_*r*_ bacteria with the lowest *J*_health_ values split and the rest of the *S*_*r*_ bacteria die.

**Step 7 (if *k* < *N*_re_, return to Step 3).**

In this instance, the specified maximum number of reproduction steps is not over; therefore the bacteria begin a new generation of a chemotactic loop.

**Step 8 (elimination dispersal).**

For *i* = 1,2,…, *S* with probability *P*_ed_, eliminate and then disperse one to a random place. If *l* < *N*_ed_, then go to Step 2, otherwise end.

## 5. Simulation and Results

The decoupled physical system and its controllers are designed and simulated with MATLAB™ Simulnik®.

The RGA method is applied on the mathematical model of the two-coupled distillation column process, and the resulted matrix is given by

(26) $$\begin{array}{c} RGA_{4\times 4}=\left[\begin{array}{cccc} -0.0429 & 0.5629 & 0.4083 & 0.0717\\ 1.4559 & 0.5558 & -0.9138 & -0.0980\\ -0.4647 & -3.1110 & 4.4832 & 0.0926\\ 0.0517 & 2.9923 & -2.9777 & 0.9337 \end{array}\right]. \end{array}$$

According to the RGA matrix the suitable input-output pairing is

(27) $$\begin{array}{c} \left(\;T_{11}-SAB\;\right),\\ \left(\;T_{30}-QE\;\right),\\ \left(\;T_{34}-RL1\;\right),\\ \left(\;T_{48}-RL2\;\right). \end{array}$$

Depending on the suitable pairing, the fitness functions of the 1st, 2nd, 3rd, and 4th input are described in (28), (29), (30), and (31), respectively.

(28) Fitness1ITSO11+ITSO31+ITSO41=16185764.6415∗λ112+−31208202.2860∗λ11∗λ21+−29085704.6263∗λ11∗λ31+−2104353.3818∗λ11∗λ41+23626420.1763∗λ212+40518661.8661∗λ21∗λ31+1053225.6938∗λ21∗λ41+17625377.5018∗λ312+1142698.7804∗λ31∗λ41+112317.1448∗λ412

(29) Fitness2ITSO22+ITSO32+ITSO42=39587638.7663∗λ122+−25965747.1866∗λ12∗λ22+−27602372.6861∗λ12∗λ32+−3313543.6120∗λ12∗λ42+6085664.7540∗λ222+12367844.1767∗λ22∗λ32+1689163.6136∗λ22∗λ42+6408628.3541∗λ322+1716766.7412∗λ32∗λ42+119596.6264∗λ422

(30) Fitness3ITSO13+ITSO23+ITSO43=32387849.9550∗λ132+−30918315.4051∗λ13∗λ23+−28156040.3138∗λ13∗λ33+−1472985.7141∗λ13∗λ43+21869260.2750∗λ232+36396234.4688∗λ23∗λ33+403039.5905∗λ23∗λ43+15212859.0003∗λ332+390512.5127∗λ33∗λ43+57318.4117∗λ432

(31) Fitness4ITSO14+ITSO24+ITSO34=40741561.5731∗λ142+−37368063.2195∗λ14∗λ24+−36883365.2610∗λ14∗λ34+−2495963.4210∗λ14∗λ44+22451663.5575∗λ242+39102720.2746∗λ24∗λ34+623243.6320∗λ24∗λ44+17326594.9351∗λ342+837511.9391∗λ34∗λ44+77118.8721∗λ442.

The BSO algorithm parameters' values which are utilized to implement an optimized decoupling network as well as an optimized BELBIC are summarized in Table 1. The resulting values of the steady state decoupling compensation elements that minimize the above fitness functions are summarized in Table 2.

The resulting best gains' values for different BELBICs that minimize the summation of the integral time-weighted squared errors (ITSEs) for different decoupled loops are presented in Table 3. The best gains' values of the designed conventional PID controllers are shown in Table 4. The PID controllers' parameters are determined utilizing the same algorithm utilized for the design of BELBICs. In this regard, proposed BSO algorithm with the same gains given in Table 1 is used to minimize the same fitness function.

**Figure 8:**
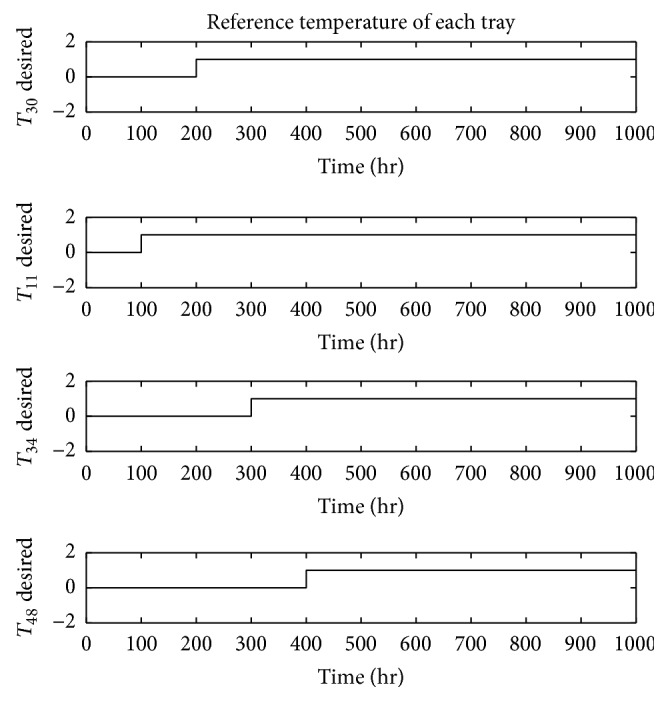
Step changes in system inputs [37].

**Figure 9:**
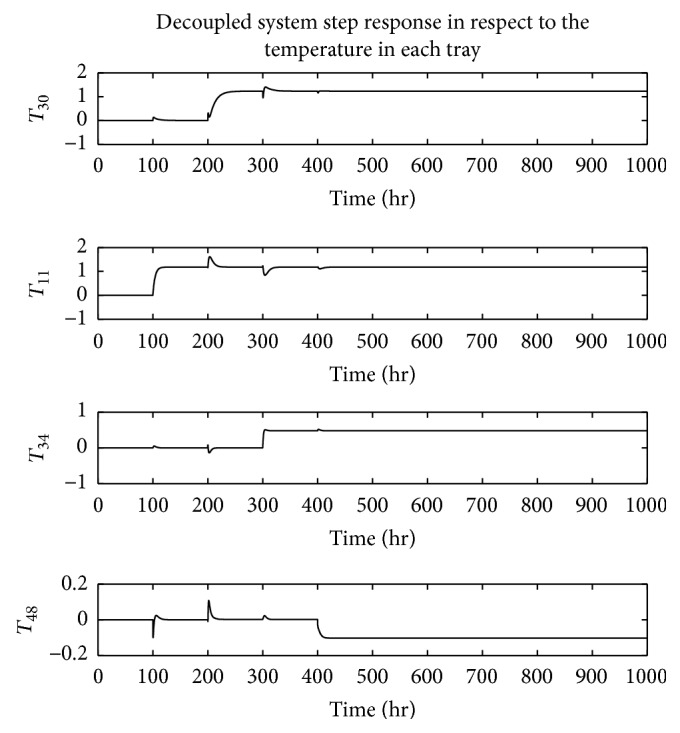
The outputs of different decoupled loops in case of no controllers.

**Figure 10:**
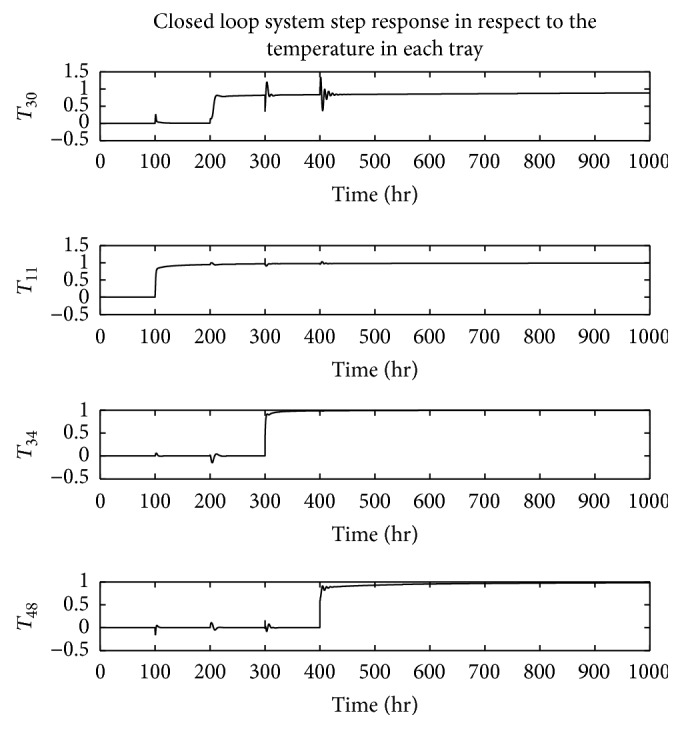
The outputs of different decoupled loops in the presence of the controller.

The dynamic behavior of the system is analyzed based on its step response based on the sequential step input shown in Figure 8. In Figure 9, the decoupled system response on the given step sequence is illustrated. As shown, the experimental results demonstrate the efficiency of the designed decoupling technique. Thus, the interrelationship between system inputs and their unpaired outputs is noticeably minimized compared to the formerly deployed techniques [11]. Accordingly, the spikes in the response of the decoupled system outputs on the unpaired inputs are significantly reduced. The step response of the designed closed loop control system is shown in Figure 10.

**Figure 11:**
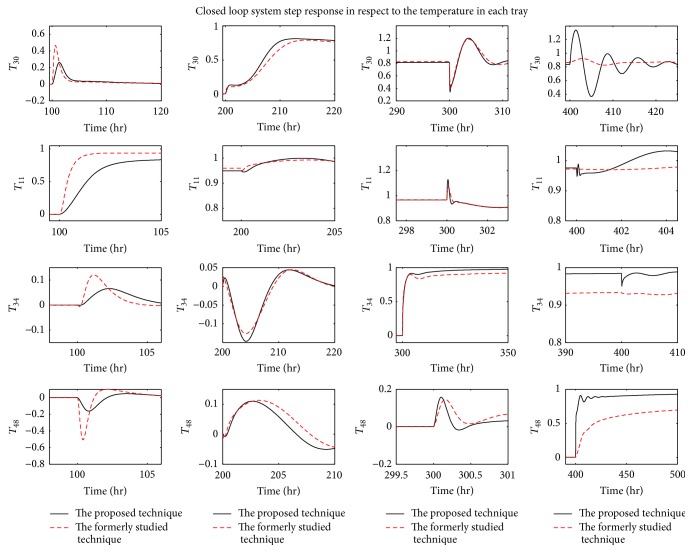
Comparison between the step response of the proposed technique and that of the formerly studied technique.

For verification purposes, the response of the designed control scheme, which is based on the minimization of ITSEs of all control loops utilizing the BSO algorithm, is compared to that of the latest research that considers the same application. It is to mention that the control scheme of the previous research is mainly based on the minimization of the ISE using the PSO algorithm [37]. In Figure 11, the proposed technique is compared to the former study in terms of step response. Moreover, the steady state errors for both controllers are stated in Table 5.

As shown, the control scheme presented in this research offers a valuable improvement in the last three control loops (*T*_11_, *T*_34_, and *T*_48_) in terms of minimizing steady state errors.

**Figure 12:**
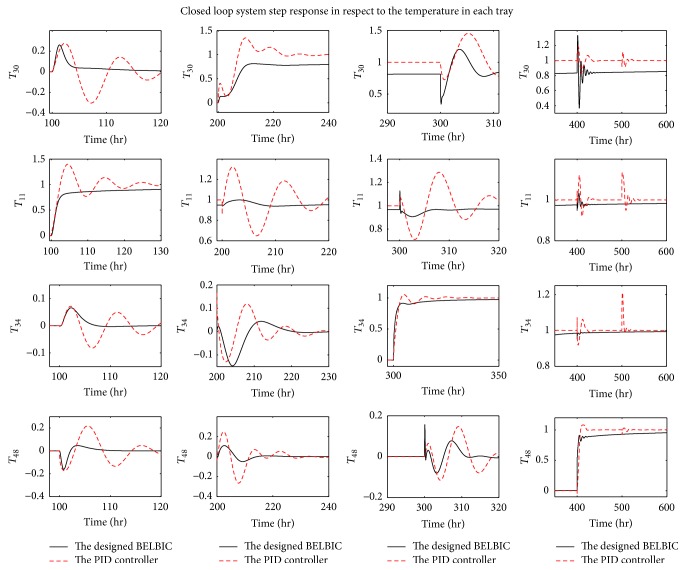
Comparison between the step response of the designed BELBIC and that of the PID controller in the presence of disturbance at *t* = 500 seconds.

The step response of the proposed BELBICs and conventional PID controllers is compared in the presence of a disturbance step with the final value of “1” at the 500th second in the first and the third decoupled loop. The simulation results are presented in Figure 12. In the figure it is clearly recognizable that the robustness of the proposed BELBICs is higher than that of the conventional PID controllers regarding the handling of the unexpected disturbance. The controlled system using BELBICs is better damped as well. On the other side, the PID controllers in all control loops achieve remarkably less steady state error.

For comparison purpose, the PID controllers are designed by utilizing the PSO technique for minimizing the summation of the integral time-weighted squared errors (ITSEs) of system control loops. This controller is to be compared with the one designed using the BSO algorithm regarding the integral time-weighted squared errors (ITSEs) for each control loop, which are listed in Table 6. The remarkable difference between both algorithms regarding ITSEs indicates that the BSO technique is more efficient in determining the global best solution in the field of MIMO control system.

## 6. Conclusions

The challenge of decoupling and controlling higher order multi-input multioutput (MIMO) process is tackled in this research. The Bacterial Swarm Optimization (BSO) technique is used to develop an optimized scheme for decoupling highly interactive 4 input/4 output two-coupled distillation column processes. The scheme consists of two stages. In the first stage, the optimum group of fitness functions is determined through the analysis of precalculated proper pairing, which is based on the derived relative gain array (RGA). The derivation of the RGA is based on the transfer function matrix of the physical process. In the second stage, the values of decoupling compensation elements (*λ*s) that minimize the interactions are estimated based on the formerly driven fitness functions. Designing the decoupled system based on the general form of the decoupling compensation matrix showed a remarkable improvement in the system dynamics compared to the utilization of the simple form of the compensation matrix. The simulation results showed the efficiency of the proposed BSO technique in estimating steady state decoupling compensation elements values. For control purpose a scheme of Brain Emotional Learning Based Intelligent Controller (BELBIC) is designed and optimized using BSO algorithm to obtain the optimal values of controllers' parameters. Furthermore, PID controllers are developed using the same optimization technique, in order to validate the robustness of the BELBIC. The robust control behavior of the designed BELBIC-based control scheme is validated in the simulation results. The BELBIC designed using the BSO algorithm showed a remarkable improvement in the transient and steady state errors of the last three control loops compared with the controller designed utilizing the PSO technique.

## Figures and Tables

**Table 1:** Gains of BSO.

Parameter	Symbol	BSO for decoupling network implementation	BSO for BELBIC implementation
Number of bacteria in the population	*S*	50	50
Dimension of search space	*p*	4	24
Maximum number of swim length	*N*_*s*_	4	4
Maximum number of chemotactic steps	*N*_*c*_	100	20
Number of reproductive steps	*N*_re_	4	2
Number of elimination dispersal events	*N*_ed_	2	2
Elimination dispersal probability	*P*_ed_	0.25	0.25
Step size	*C*(*i*)	0.05	0.05
Cognitive factor	*C*_1_	1.2	1.2
Social acceleration factors	*C*_2_	0.5	0.5
Momentum/inertia	*w*	0.9	0.9

**Table 2:** Resulting values of steady state decoupling compensation elements based on BSO.

Fitness function	Final value of fitness function	Values of steady state decoupling elements (*λ*s)			
Fitness_1_	9.8506	λ_11_ = 0.2453	λ_21_ = −0.4755	λ_31_ = 0.7210	λ_41_ = 0.8564
Fitness_2_	0.5941	λ_12_ = −0.0195	λ_22_ = −0.1090	λ_32_ = −0.0967	λ_42_ = 1.1934
Fitness_3_	7.6138	λ_13_ = −0.0481	λ_23_ = 0.6249	λ_33_ = −0.7904	λ_43_ = −0.1218
Fitness_4_	0.3040	λ_14_ = −0.0026	λ_24_ = 0.1492	λ_34_ = −0.1790	λ_44_ = 0.3273

**Table 3:** The proper gains of the BELBICs optimized by the BSO for different loops.

Loop	Gains					
	α	β	*K*	*K*_*p*_	*K*_*i*_	*K*_*d*_
(QE, *T*_30_)	0.2321	0.8914	−0.4830	3.4765	0.0028	−0.6471
(SAB, *T*_11_)	0.3418	−1.3651	0.3936	−21.5658	−1.0949	−0.7771
(RL1, *T*_34_)	87.3587	−8.9268	−0.1020	29.4672	17.3183	14.6593
(RL2, *T*_48_)	17.4282	22.6201	0.2650	−62.2460	−0.6042	10.8162

**Table 4:** The proper gains of the PID controllers optimized by the BSO for different loops.

Loop	Gains		
	*K*_*p*_	*K*_*i*_	*K*_*d*_
(QE, *T*_30_)	1.9662	0.3524	0.7445
(SAB, *T*_11_)	1.0643	1.9718	0.2621
(RL1, *T*_34_)	1.6276	1.4202	−0.0366
(RL2, *T*_48_)	0.0784	−3.0889	0.8859

**Table 5:** The steady state errors for all loops after control.

Loop	Steady state errors	
	Former technique	Proposed technique
(QE, *T*_30_)	0.087510	0.1189
(SAB, *T*_11_)	0.019915	0.0117
(RL1, *T*_34_)	0.036152	0.0041
(RL2, *T*_48_)	0.189710	0.0207

**Table 6:** Comparison between PID controllers designed by PSO and BSO algorithms regarding integral time-weighted squared error ITSE.

Loop	ITSE	
	PSO technique	BSO technique
(QE, *T*_30_)	11.5703	3.2552
(SAB, *T*_11_)	0.4473	1.8333
(RL1, *T*_34_)	2.7937	1.8592
(RL2, *T*_48_)	11.8501	5.9165

*Summation*	*26.6614*	*12.8642*

## List of Symbols

QE: Heat input to the reboiler
SAB: Vapor flow rate in the vapor transfer line
RL1: Reflux ratio in the main column
RL2: Reflux ratio in the second column
*T*_11_: Temperature at the 11th tray
*T*_30_: Temperature at the 30th tray
*T*_34_: Temperature at the 34th tray
*T*_48_: Temperature at the 48th tray
*G*(*s*): Transfer function matrix of the process in *s* domain
*γ*_*ij*_: Relative gain between the output “*i*” and input “*j*”
Λ_ss_: Steady state decoupling compensation matrix
*λ*_*ij*_: Decoupling compensation element between the output “*i*” and input “*j*”
*Y*(*s*): Output vector of the process
*W*(*s*): Input vector of the decoupling network
MO: The model output of Brain Emotional Learning Based Intelligent Controller (BELBIC)
*O*_*i*_: Orbitofrontal cortex node
*A*_*i*_: Amygdala node
*S*_*i*_: The *i*th sensory input
*G*_*A*_: Plastic connection weights of the amygdala
*G*_*O*_: Plastic connection weights of the orbitofrontal cortex
*α*: The amygdala learning rate
*β*: The orbitofrontal cortex learning rate
Rew: The reward signal
*e*: Error signal
*K*, *K*_*p*_, *K*_*i*_, *K*_*d*_: Controller parameters
*T*_30_ desired: Reference signal for output *T*_30_
*T*_11_ desired: Reference signal for output *T*_11_
*T*_34_ desired: Reference signal for output *T*_34_
*T*_48_ desired: Reference signal for output *T*_48_
*p*: Dimension of search space
*S*: The number of bacteria
*S*_*r*_: The number of bacteria splits per generation
*N*_*c*_: The number of chemotactic steps
*N*_*s*_: Limits of the length of a swim
*N*_re_: The reproduction steps number
*N*_ed_: The amount of elimination-dispersal events
*P*_ed_: Elimination-dispersal probability
*C*(*i*): The bacteria step size length
Delta: The direction of each bacteria
*C*_1_: Weight of local information
*C*_2_: Weight of global information
*R*_1_, *R*_2_: Two random numbers.
